# Food Insecurity, Safety Nets, and Coping Strategies during the COVID-19 Pandemic: Multi-Country Evidence from Sub-Saharan Africa

**DOI:** 10.3390/ijerph18199997

**Published:** 2021-09-23

**Authors:** Shouro Dasgupta, Elizabeth J. Z. Robinson

**Affiliations:** 1 Centro Euro-Mediterraneo sui Cambiamenti Climatici (CMCC), 30175 Venice, Italy; 2 RFF-CMCC European Institute on Economics and the Environment (EIEE), Centro Euro-Mediterraneo sui Cambiamenti Climatici (CMCC), 30175 Venice, Italy; 3 Department of Environmental Sciences, Informatics and Statistics, Università Ca’ Foscari Venezia, 30175 Venice, Italy; 4 Grantham Research Institute on Climate Change and the Environment, London School of Economics and Political Science (LSE), London WC2A 2AE, UK; e.j.z.robinson@lse.ac.uk

**Keywords:** COVID-19, food insecurity, multi-country, socioeconomic determinants

## Abstract

The COVID-19 pandemic has affected food security across the world. As governments respond in different ways both with regards to containing the pandemic and addressing food insecurity, in parallel detailed datasets are being collected and analysed. To date, literature addressing food insecurity during the pandemic, using these datasets, has tended to focus on individual countries. By contrast, this paper provides the first detailed multi-country cross-sectional snapshot of the social dimensions of food insecurity during the COVID-19 pandemic across nine African countries (Chad, Djibouti, Ethiopia, Kenya, Malawi, Mali, Nigeria, South Africa, and Uganda). Econometric analysis reveals that female-headed households, the poor, and the less-formally educated, appear to suffer more in terms of food insecurity during this global pandemic. Importantly, our findings show that the negative consequences of the pandemic are disproportionately higher for lower-income households and those who had to borrow to make ends meet rather than relying on savings; impacts are country-specific; and there is considerable spatial heterogeneity within country food insecurity, suggesting that tailored policies will be required. These nine countries employ both food and cash safety nets, with the evidence suggesting that, at least when these data were collected, cash safety nets have been slightly more effective at reducing food insecurity. Our results provide a baseline that can be used by governments to help design and implement tailored policies to address food insecurity. Our findings can also be used as lessons to reshape policies to tackle the heterogeneous impacts of climate change.

## 1. Introduction

The COVID-19 pandemic has had significant negative impacts on people’s incomes and livelihoods across the world. According to the World Bank, global GDP per capita was expected to decline by 6.2% in 2020, while Sub-Saharan Africa and South Asia were expected to suffer 5.3% and 3% declines, respectively [1]. This loss of income, combined with the associated use of lockdowns to control the spread of the virus, has had severe repercussions on access to food, and food security more broadly, particularly in lower-income countries [2,3,4].

Food security is defined by the FAO as existing when “all people, at all times, have physical and economic access to sufficient, safe and nutritious food that meets their dietary needs and food preferences for an active and healthy life” [5]. As such, the focus is on whether there is sufficient food, in the right location, and whether people can afford to purchase it. COVID-19 has affected access to food across most, if not all, the dimensions of food security, and the literature on why and to what extent this was the case has grown rapidly. For example, ref. [6] highlights the impact of COVID-19 on falling incomes, poverty, and the ability of households to purchase food; agricultural production, supply chain disruption, and trade restrictions; and increased deficiencies in key micro-nutrients. Others similarly focus on disruptions to agricultural sectors and particularly supply chains, resulting in increased food prices and/or food shortages in low- and middle-income countries (LMICs) [7,8,9].

Governments, even outside of crises, have long employed safety nets as part of a suite of social protection measures designed to ameliorate poverty and food insecurity [10]. Perhaps not surprisingly therefore, safety nets have been highlighted as particularly important during the pandemic [11], especially in countries where hunger and malnutrition have increased due to COVID-19 [12]. Authors highlight the importance of both cash and food transfers. Yet, safety nets too have been negatively affected by the pandemic, including those explicitly linked to food [13].

It is only recently that data are becoming available to quantify and build a picture of food insecurity in low- and middle-income countries (LMICs) during the COVID-19 pandemic. For some African countries, comparable data are available to explore how food insecurity has changed due to the pandemic. For example, ref. [14], using pre-pandemic data collected using a face-to-face survey and follow-up phone survey data collected during the pandemic, found that households’ experiences of food insecurity have increased by between 6 and 15 percent. Though the authors highlighted the likely importance of social safety nets, their role in reducing food insecurity is not addressed explicitly. A study of households in Kenya and Uganda found that food security had worsened due to the pandemic, with households changing their dietary patterns in response to a loss of income. Both governments responded in various ways, with the Ugandan government providing a food safety net for vulnerable workers [15].

By contrast, panel data collected in Addis Ababa, Ethiopia, before and during the pandemic, found little change in food insecurity [16]. Ref. [15] used an online questionnaire to collect data from 313 and 129 respondents in Kenya and Uganda, respectively. For food insecurity, the questionnaires followed the FIES survey module (FAO 2016), which asks eight questions on food insecurity before and during the COVID-19. These questions cover uncertainty about food supply, food variety and quality, insufficient food intake, and experiencing hunger among household members.

To date, the literature addressing food security and COVID-19 in sub-Saharan Africa has focused primarily on individual country studies, making use of panel data as they become available, with little attention to cross-country studies. To fill this gap, in this paper, we use unique micro-surveys from nine African LMICs to take a first look at the food insecurity situation in the context of the pandemic. To the best of our knowledge, importantly, this paper provides the first cross-sectional analysis of food insecurity during the COVID-19 pandemic across multiple African countries. We focus particularly on the socioeconomic dimensions of food insecurity, presenting a snapshot of food insecurity differentially experienced by households during a specific global pandemic. We highlight the role that safety nets, both food and cash, play in food security, and, as such, our findings can help to guide governments as to where best to target efforts to reduce food insecurity that households are experiencing.

In the next section, we present our methodology and data sources. In Section 3, we present key descriptive statistics on food security and governments responses to the pandemic, and for the countries in our sample that have sufficiently detailed data, we empirically explore the socioeconomic determinants of food insecurity, including differential impacts of gender, age, income, safety nets, reliance on savings, and increase in the prices of major food items. Section 4 discusses these findings and Section 5 concludes.

## 2. Methods and Data

Since the beginning of the pandemic, a number of lower-income countries, many in Africa, have conducted High Frequency Phone Surveys (HFPS) of households linked to ongoing panel micro studies. Most of these surveys have been conducted by the national statistical institutes in collaboration with the World Bank. However, individual countries have also added COVID-19 modules to their existing household surveys (for example, South Africa). The main aim of these surveys is to track and monitor the socioeconomic impacts of the pandemic with a focus on employment, income (wages and business revenue), health, education, food security, and coping strategies, including safety nets. Given the social distancing measures implemented by governments across the world, face-to-face surveys that would generally be used to conduct population-based surveys have had to be suspended. Phone surveys do not require in-person interviews; they can be used to obtain similar information at a relatively low cost; and they offer flexibility in terms of sampling and questionnaire design. However, phone surveys have some limitations that may induce bias in the information obtained, including: (i) study attrition or non-response of respondents from the original/base survey, especially if there is a significant gap between the last in-person survey and the phone survey; (ii) selection bias induced by mobile phone ownership; (iii) differential mobile phone coverage within countries and demographic groups; (iv) that it may not be possible to verify accuracy of answers commonly used in face-to-face interviews, such as vaccine cards or utility bills; and (v) some topics such as mental health and sexual behaviour may be difficult to cover in a phone survey. Despite the concerns regarding phone coverage, the share of households with contact information for at least one household member or a reference person in the sample of countries ranges from 79.2 to 99.2%. Finally, there is no evidence to suggest that attrition is significant because adequate measures to replicate the sampling characteristics of the previous population-survey have been taken. Sample sizes vary across the nine countries. As examples, 39% of the respondents from the Nigeria GHS-panel in 2018–2019 survey were interviewed for the COVID-19 round; a sub-sample of 48% from the 2018–2019 Ethiopia Socioeconomic Survey (ESS); and 54.4% from the Malawi Integrated Household Panel Survey (IHPS), conducted in 2019.

Our motivation behind the selection of these nine countries is based on data availability. At the time of writing, though surveys in some other countries were being undertaken, either they were not complete or the data had not yet been made available. These nine countries comprise 49% of the total population of sub-Saharan Africa. They also consist of at least one country from each of the regions in sub-Saharan Africa: Chad from Central Africa; Djibouti, Ethiopia, Kenya, Malawi, and Uganda from Eastern Africa; Mali and Nigeria from Western Africa; and South Africa from Southern Africa. Finally, these countries are also representative of the different income groups, according to the World Bank country classifications by income level: five are low-income (Chad, Ethiopia, Malawi, Mali, and Uganda); three are lower-middle income (Djibouti, Kenya, and Nigeria); and one is upper-middle income (South Africa). The summaries of the datasets that we use in this study are provided in the following sub-section.

### 2.1. Individual Country Food Insecurity Data Sources in Selected Countries

Chad: With support from the World Bank, Institut National de la Statistique, des Etudes Economiques et Démographiques (INSEED) carried out the Chad COVID-19 impact monitoring survey. The survey’s sample consists of 1748 households and was drawn from the Enquête sur la Consommation des Ménages et le Secteur Informel au Tchad (Ecosit 4), conducted in 2018–2019.

Djibouti: The National Institute of Statistics of Djibouti, with support from the World Bank, launched the COVID-19 National Panel Phone Survey in July 2020 to study the impacts of the pandemic. The survey consists of 1486 households drawn randomly from the social registry data restricted to urban households.

Ethiopia: To monitor the impacts of the pandemic on households, a sub-sample of 3249 households was drawn from the sample of households interviewed in the 2018–2019 round of the Ethiopia Socioeconomic Survey (ESS) in collaboration with the World Bank.

Kenya: This survey is being conducted by the World Bank in collaboration with the Kenya National Bureau of Statistics (KNBS) and the University of California Berkeley. The first sample is a randomly drawn subset of all households that were part of the 2015–2016 Kenya Integrated Household Budget Survey (KIHBS) Computer-Assisted Personal Interviewing (CAPI) pilot. It includes information on household background, service access, employment, food security, income loss, transfers, health, and COVID-19 knowledge for 4457 households.

Malawi: A High-Frequency Phone Survey COVID-19 (HFPS COVID-19) was carried out by the National Statistical Office (NSO) in collaboration with the World Bank and the United States Agency for International Development (USAID). A total of 1729 households drawn from the Integrated Household Panel Survey (IHPS 2019) were interviewed with a 95% response rate for the first round. The aim of the survey was to assess the socioeconomic impact of the pandemic with a focus on behaviour and social distancing, access to basic services, employment and income, food, security, social safety nets, and agriculture.

Mali: The COVID-19 Panel Phone Survey of Households 2020 was implemented by the National Statistical Office (INSTAT) in collaboration with the World Bank in May 2020. The survey consists of 1809 households and provides information on behaviour and social distancing, access to basic services, employment and income, prices and food security, income loss, and social safety nets.

Nigeria: The Nigeria COVID-19 National Longitudinal Phone Survey (COVID-19 NLPS) was implemented by the National Bureau of Statistics in collaboration with the World Bank. The survey was conducted on a nationally representative sample of 1950 households between 20 April and 11 May 2020 drawn from wave 4 of the General Household Survey—Panel (GHS-Panel) in Nigeria.

South Africa: These data come from the the National Income Dynamics Study-Coronavirus Rapid Mobile Survey (NIDS-CRAM). The aim of this survey is to investigate the socioeconomic impacts of the national lockdown in South Africa in March 2020, and the social and economic consequences of the global coronavirus pandemic. NIDS-CRAM is based on a sub-sample of adults from households in the National Income Dynamics Study (NIDS) Wave 5 (2017) and provides data on 7074 completed interviews.

Uganda: The High-Frequency Phone Survey was launched in June 2020 by the Uganda Bureau of Statistics (UBOS) with support from the World Bank. The survey tracks the impacts of the pandemic. A total of 2259 households were interviewed from the Uganda National Panel Survey (UNPS) 2019–2020.

We use these high-Frequency Phone Survey data on COVID-19 to track the impacts of the pandemic within and across countries. We present and analyse data from nine countries. Specifically, we look at the following four indicators of household food insecurity from the survey questionnaire:

1. Have you or any other adult in your household had to skip a meal?
2. Did the household go without eating for a whole day in the last 30 days?
3. Did you or any other adult in your household run out of food?
4. Were you or any other member in your household hungry but did not eat?

### 2.2. Econometric Methodology

We use a Probit regression (Equation (1)) to investigate the socioeconomic determinants of food insecurity during the early months of the pandemic for each country using household-level data:

(1) $$P_{i}=Prob\left(y=i|X\right)=\frac{exp(X\beta_{i})}{1+\sum_{k=1}^{m}exp\left(X\beta_{i}\right)}$$

where i=1 if a household responds *yes* to the food security-related questions mentioned above. We control for socioeconomic and demographic characteristics such as education, gender, and age of household head, log of pre-lockdown household income, and poverty status of households in the case of Chad, Djibouti, and Mali (as income data are not available for these countries). There is evidence in the literature that female-headed households tend to be at higher risk of food insecurity compared with the male-headed ones, due to the fact that women often have poorer access and control of resources [17,18,19,20], and so we anticipate the same for our data. Given evidence in the literature of a positive impact of education on income [21,22,23] and health [24,25], we expect households with higher educated heads to have lower chances of suffering from food insecurity. A household’s ability to afford food is likely to be a strong determinant of food security. We therefore control for pre-pandemic income and expect that relatively higher income households will have a lower probability of food insecurity, while households that are considered poor are likely be at a higher risk of food insecurity. Because affordability of food is likely to be a strong determinant of food security, we include an additional dummy variable indicating whether the prices of major food items increased during the pandemic.

The data allow us to control for whether a household has received a safety net in the form of cash or food for all the countries except Kenya and Uganda, for which such a breakdown is not available. For these two countries, we therefore can only account for whether a household has benefited from any safety net. There is considerable literature on the choice of appropriate safety nets, with heterogeneous evidence on their impacts [26,27]. We also control for two important coping strategies that households have adopted during the pandemic: reliance on savings; and borrowing from friends, family, and non-government organisations. Finally, we include location (sub-national) fixed-effects controlling for unobserved heterogeneities such as sub-national level supply chain disruptions and differences in government restrictions. We use the sampling survey weights provided in the HFPS datasets.

## 3. Findings

In the initial stages of the pandemic, it is difficult to know the extent to which cases of COVID-19 and deaths related to COVID-19 have been accurately reported [28]. This caveat notwithstanding, from the World Bank’s 2020 report [1] a common narrative emerges of countries affected by illness, death, reduced economic growth or even economic contraction, and a loss of income and livelihoods more broadly. Here, we first document how governments in these countries have responded to the pandemic, in particular, exploring the extent to which populations in these countries have been subjected to restrictions, and the consequent disruptions on economies, on jobs, and on livelihoods. We identify the efforts governments have taken to ameliorate the worst impacts of these restrictions. We then present individual country dimensions of food insecurity for selected countries for which data are available. Finally, we present the results of our regression analyses that explore the socioeconomic determinants of food security in these countries during the 2020 pandemic.

### 3.1. Government Responses to the Pandemic

Governments across the world have implemented various measures to contain the spread of the COVID-19 pandemic. Many of these measures, such as closing international borders, dusk-to-dawn curfews, the closure of markets, and other “lockdown” measures, whilst reducing the spread of the disease, have had a negative impact on livelihoods, and particularly food security, due to a combination of both food shortages and high prices [4]. Most governments have therefore also intervened to reduce these negative impacts, primarily by expanding existing or introducing new safety nets, whether cash or food.

#### 3.1.1. Government Restrictions and Consequent Disruptions

**Figure 1:**
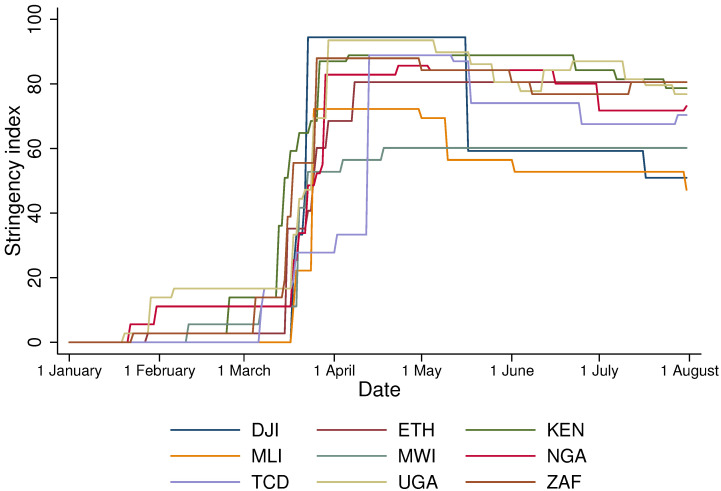
Stringency of government responses according to OxCGRT.

The Oxford COVID-19 Government Response Tracker (OxCGRT) tracks government policies and interventions including school closings, travel restrictions, bans on public gatherings, emergency investments in healthcare facilities, new forms of social welfare provision, contact tracing, and other interventions to contain the spread of the virus and augment health systems [29]. We report the stringency of the containment measures in Figure 1. The data suggest that the stringency of the government responses reached their peaks during March and April, with only Djibouti and Mali relaxing the restrictions relatively early at the end of July. In August 2020, most of the sample countries continued to impose considerable restrictions. While the severity of government restriction could affect food insecurity, sub-national level data on these restrictions are not available, and, as a result, government restrictions could not be included in our econometric analysis.

Given these restrictions, it is perhaps not surprising that across the nine countries, access to food has been disrupted, whether due to loss of income, disrupted food markets, or increased food prices. In Chad, for example, the lockdown imposed by the government to stem the spread of the virus disrupted food markets, causing the price of millet, a key staple, to rapidly increase [30]. Chad already had one of the highest levels of hunger in the world, with two-thirds of the population living in severe poverty before the pandemic [31]. Two-thirds of the survey respondents in Chad reported a loss in total income due to the economic slowdown as a result of the pandemic, largely driven by loss of family enterprise revenue (63% of family enterprises reported a decline in revenue); 20% of the respondents lost their jobs due to pandemic and the associated lockdown. The World Bank estimates that Chad’s GDP will grow by 0.8% in 2020, much lower than the 2019 growth rate of 3.2% [1].

According to the survey data, 19% of the breadwinners among the respondents in Djibouti lost their jobs due to the pandemic, while approximately 45% and 36% received either no payment or partial wages, respectively, during the lockdown. The World Bank projected a 1.3% GDP growth in 2020 in contrast to 8.4% in 2018 and 7.5% in 2019 [1]. The World Bank estimated that the GDP of Mali would shrink by 2% in 2020, in contrast to growth rates of 4.7% and 5% in 2018 and 2019, respectively [1].

In Kenya, the government implemented a wide range of measures to reduce the spread of the virus that directly affected the transport of food, including the closure of international borders and produce markets, which are critical for food distribution [32]. According to the survey, 5.3% of the workers reporting being laid off while 4% of the businesses laid off at least one employee since the outbreak. Kenya’s GDP was expected to contract by 1% in 2020, compared with the growth rates of 6.3% and 5.4% in 2018 and 2019, respectively [1].

More than 40% of the survey respondents in Nigeria reported losing their jobs as a result of COVID-19. More broadly, income losses among households have been widespread in the country, with 16 out of the 37 states in the country reporting that more than 80% of the households surveyed had their total income reduced as a result of the pandemic. According the World Bank, the Nigerian economy was projected to contract by 4.1% in 2020 [1].

Data from the South Africa survey suggest an 18% decline in employment between February and April 2020, of which two-thirds were women. According to the NIDS-CRAM survey, the proportion of adult income earners in February declined by 33%. The South African economy was estimated to shrink by 7.8% in 2020, one of the highest in the region [1]. In Uganda, almost 15% of the respondents reported that they had stopped working in the week following the imposition of the lockdown; since the pandemic began, 87% of households reported reduced income or no income from at least one of their sources of livelihood. Despite these difficulties, Uganda’s GDP was still expected to grow by 2.9% in 2020 (compared with the 6.2% and 6.8% growths in 2018 and 2019) [1].

Food security in several countries has been negatively affected due to both COVID-19 and other shocks, such as political unrest in Ethiopia and weather conditions in Malawi. In Ethiopia, 13% of respondents reported losing their jobs since the outbreak (18% in urban areas and 10% in rural); 55% of respondents reported either a reduction (51%) or a total loss (4%) in income. Despite these impacts, the Ethiopian economy was expected to grow by 6.1% in the 2019–2020 fiscal year [1], well below initial projections. In Malawi, some regions experienced excess rain, and others insufficient rain, in parallel with COVID-19 restrictions that drove up unemployment, and also coinciding with the January–February lean season [33]. Indeed, 9% of respondents stopped working in the week prior to the interview, and more than 88% of businesses reported either a drop in revenue or earning no revenue since the pandemic began. The economy of Malawi was projected to grow by 1% in 2020, significantly lower than the initial projection of 4.8% and growth rates of 3.2% and 4.4% in the previous two years [1]. As a consequence, COVID appears to be reversing the positive improvements in food security and general nutrition that the country experienced in the years before the pandemic [34]. The extent of this reversal is likely to be revealed as further waves of data are collected.

#### 3.1.2. Safety Net Responses

**Figure 2:**
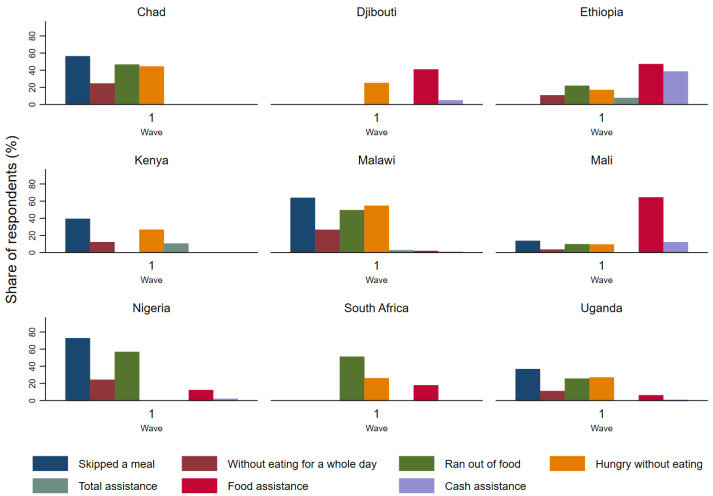
Food insecurity and safety nets at the beginning of the pandemic.

Government safety nets tend to be either in the form of cash transfers, which involve transferring small sums of cash to households, thereby redistributing wealth, protecting lower-income households against income shocks, and enabling households to improve their food security; or food assistance, which involves the provision of food, either directly, or through instruments such as food stamps or coupons that may be used to purchase food to assure a minimum level of food consumption (Figure 2). In response to reduced food security due to the pandemic, primarily caused by higher prices of staples and lower incomes due to job losses, governments and international organisations have already distributed, or committed to distributing, additional cash and food benefits. For example, in Chad, the World Bank funded the distribution of 437,000 food kits to the most vulnerable, and seeds and equipment to smallholder farmers [35]. Unconditional cash transfers have been included in the FAO’s COVID-19 business continuity plan [30]. At the time of writing, two waves of the World Bank survey had been undertaken for Djibouti (July and September/October 2020). The second wave revealed that 27% of respondents received government food stamps, 11% food assistance, and 4% cash transfers (this compares with 27%, 14%, and 5% in the first wave); 28% of households had cut the size of their meals, and 14% had skipped meals, though just 4% went a whole day without food [36]. In Ethiopia, just 8% of respondents had received government assistance, primarily free food (47%) or a direct cash transfer (39%) [37]. In Kenya, to mitigate the negative impact of COVID-19 restrictions, the government provided direct cash transfers to the poorest households through a scheme called GiveDirectly [32].

### 3.2. Indicators of within Country Food Insecurity

We present descriptive statistics for key food insecurity indicators, using the HFPS datasets. These indicators can be interpreted in part as households’ coping strategies, with households reducing their expenditure on food by reducing the quantity of the food they consume. There is additional evidence that households also reduce the quality of the food consumed [13,15]. Though we have data for nine countries, not all nine have tracked all four of the indicators: “skip a meal”; “go without eating for a whole day”; “ran out of food”; and “hungry but did not eat”.

Households in Chad, Malawi, and Nigeria appear to have experienced the highest proportion of respondents experiencing particularly extreme food insecurity, going “without eating for a whole day”, and more than half of respondents reported skipping a meal in the 30 days prior to the interview. The data (Figure 2 and Table 1) suggest that households in Chad, Malawi, and Nigeria experienced the highest share of households suffering from food insecurity, while the reported level of food insecurity in Mali has been comparatively lower.

**Figure 3:**
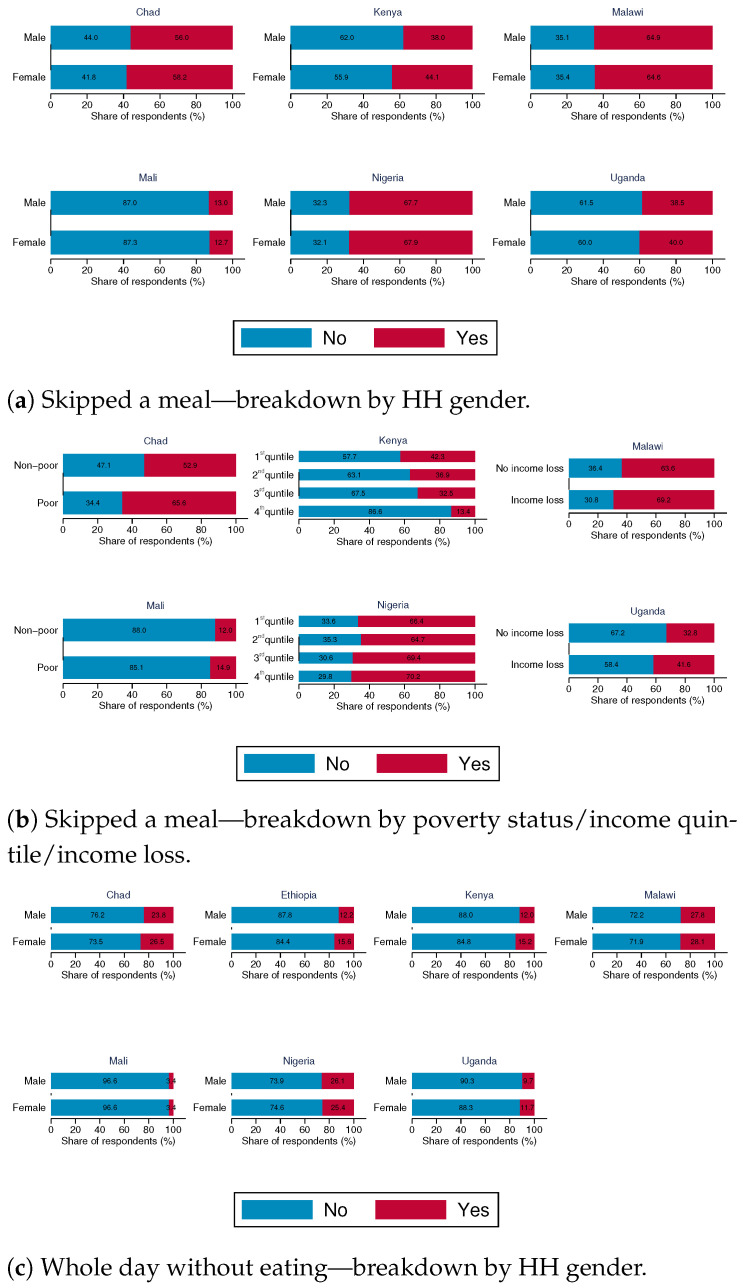

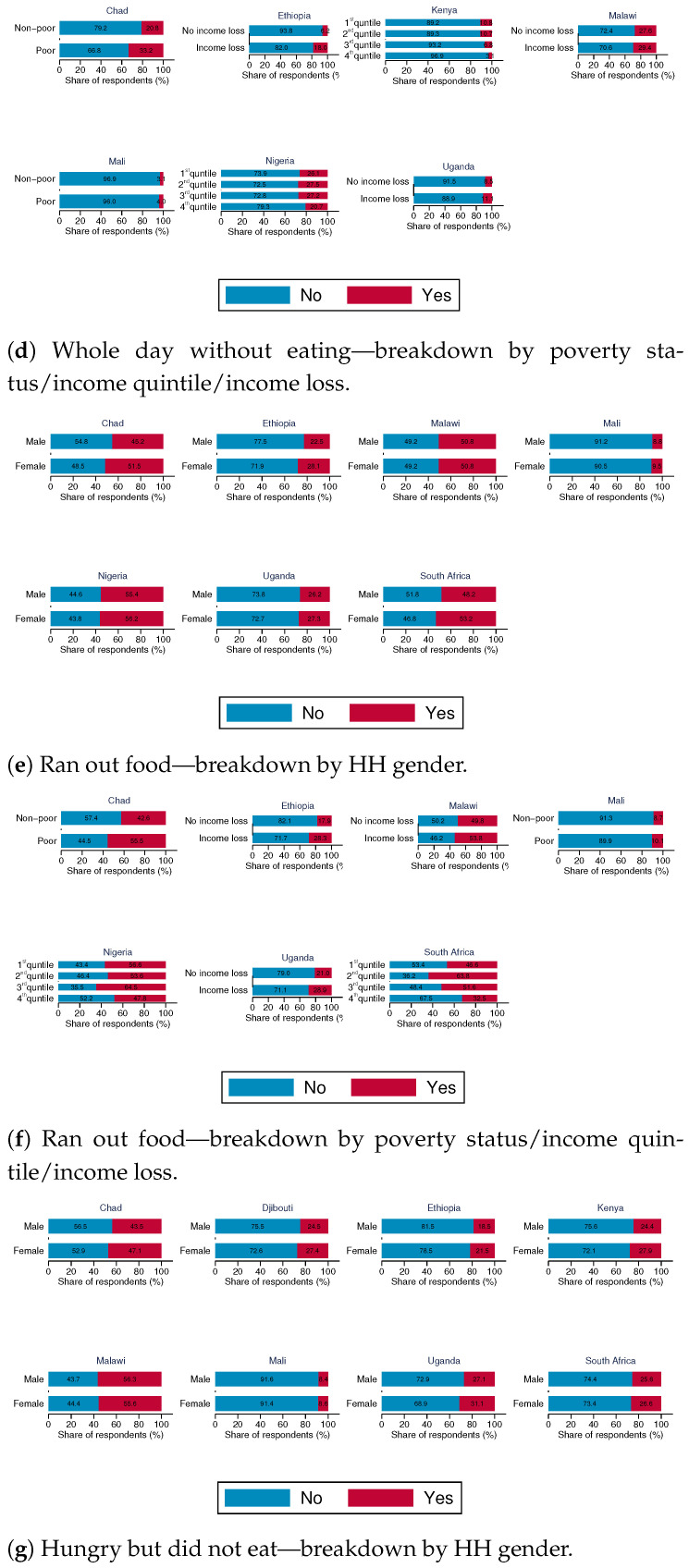

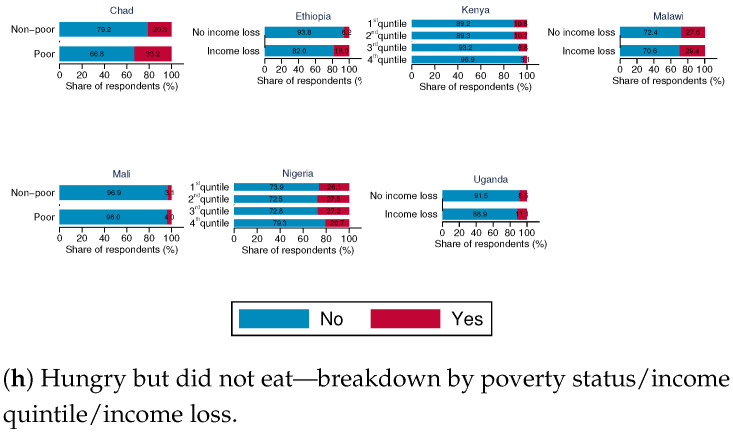
Food insecurity—breakdown by gender of household head and poverty status/income quintile/income loss.

We desegregate the responses to food insecurity indicators by gender of the household head and poverty status of households/income quartiles/whether households suffered a loss in income since the pandemic started (depending on data availability). The data (Figure 3) show that the incidence of food insecurity is higher among households with a female head compared with those with a male head. As expected, households that are considered poor/have suffered a decline in income during the pandemic and those in the first and second quartiles for total income are also more likely to suffer from food insecurity.

**Figure 4:**
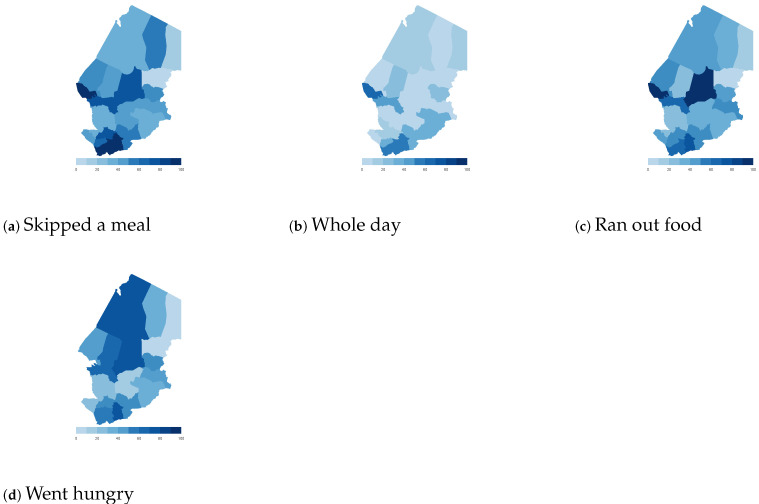
Chad—share of households.

**Figure 5:**
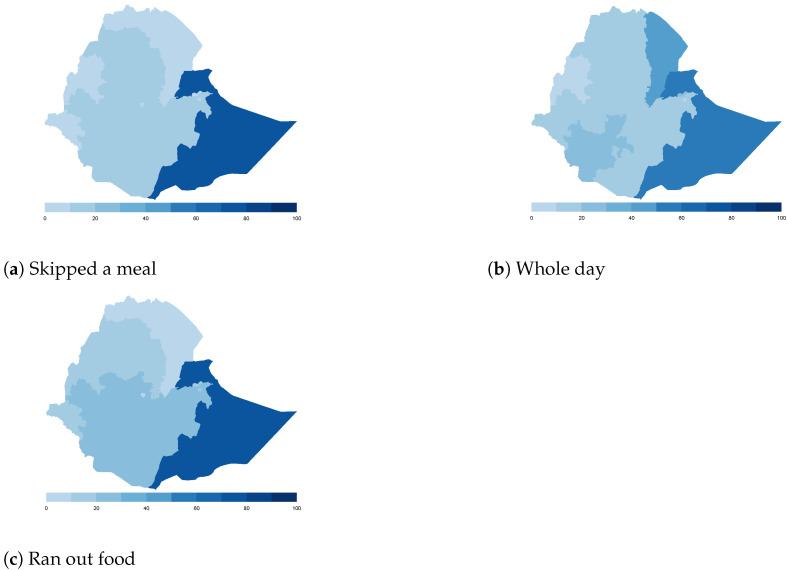
Ethiopia—share of households.

**Figure 6:**
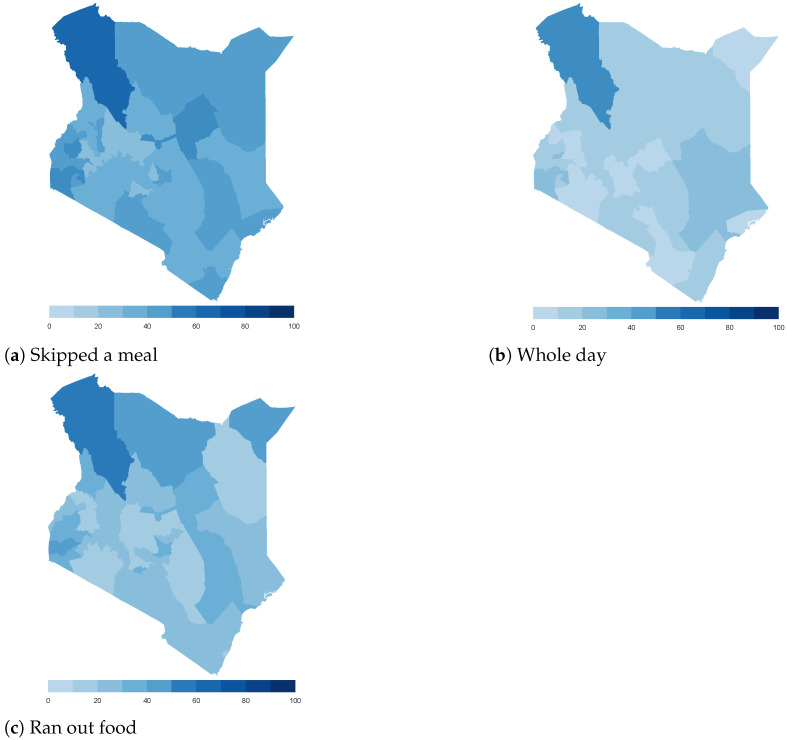
Kenya—share of households.

**Figure 7:**
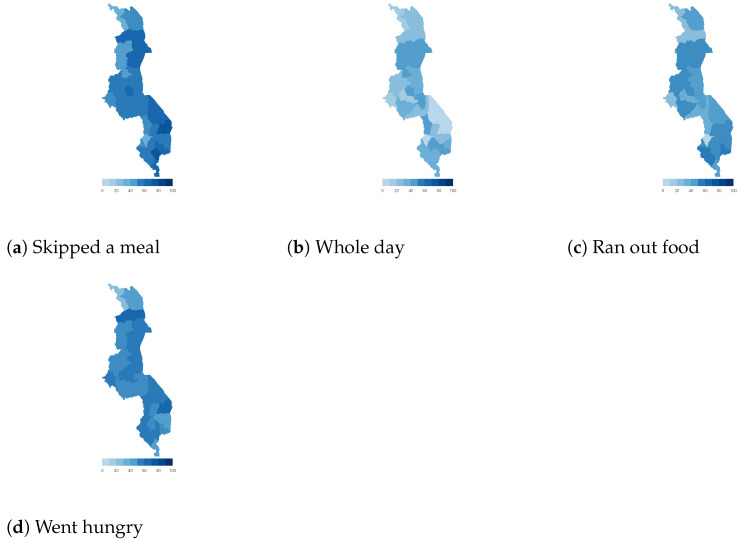
Malawi—share of households.

**Figure 8:**
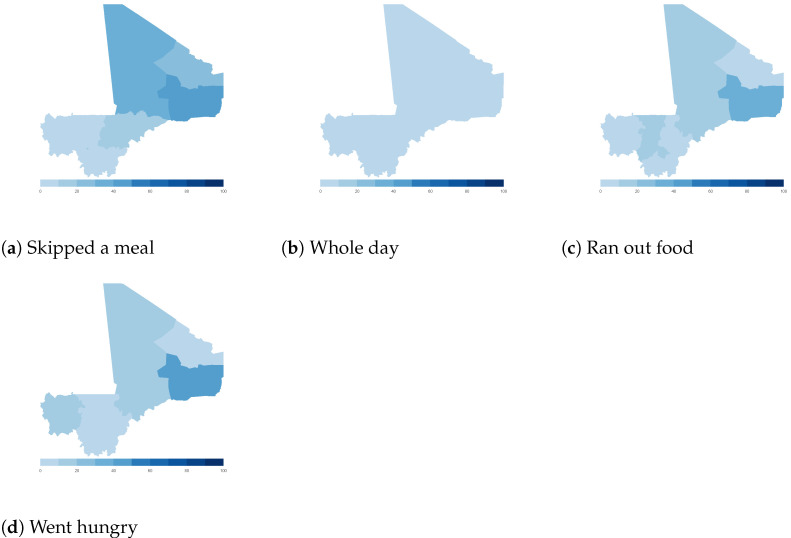
Mali—share of households.

**Figure 9:**
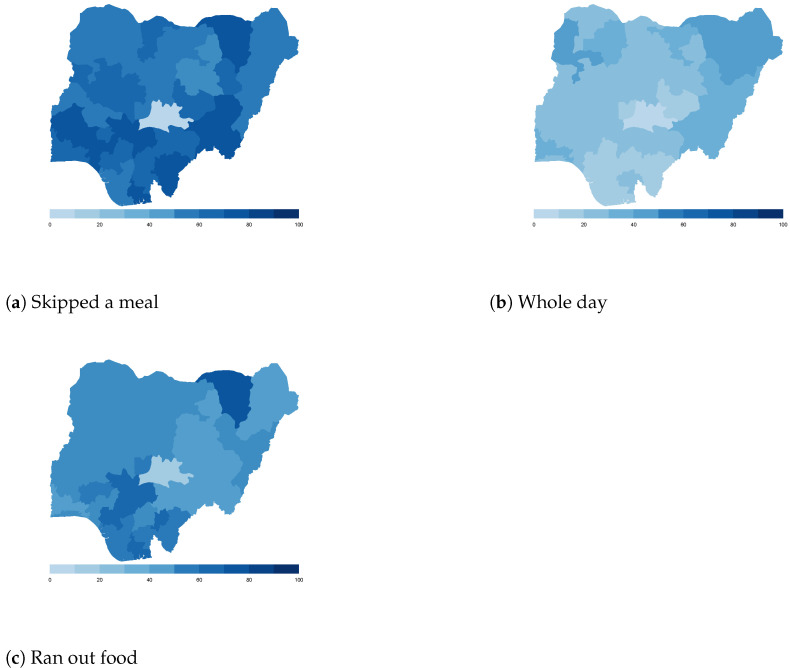
Nigeria—share of households.

**Figure 10:**
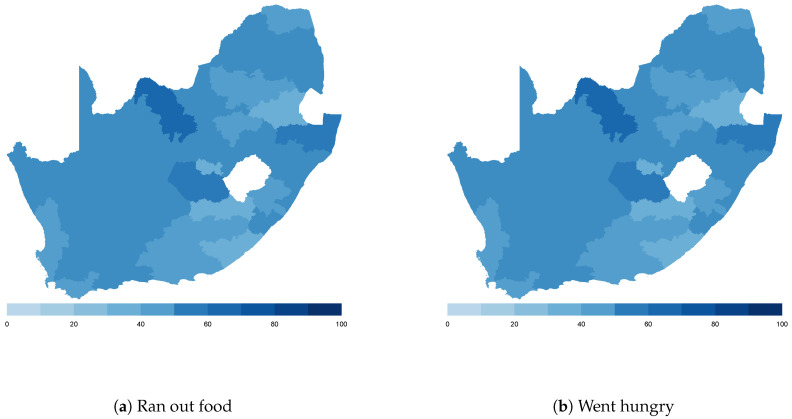
South Africa—share of households.

**Figure 11:**
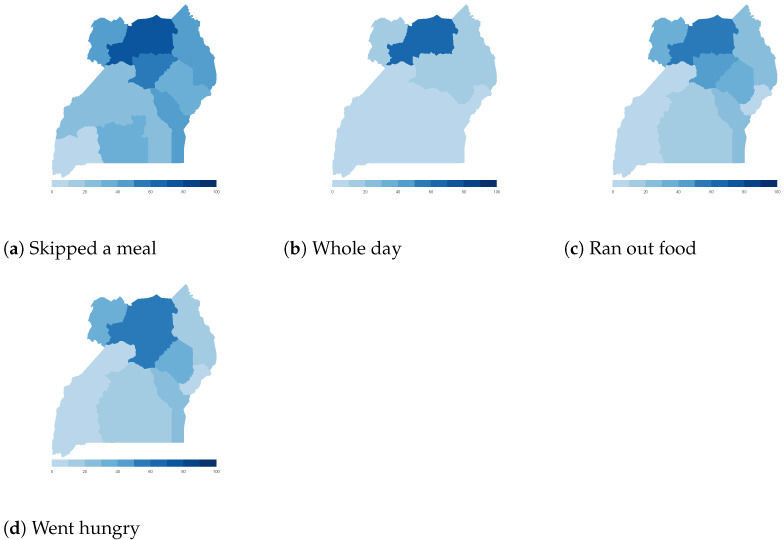
Uganda—share of households.

Finally, we map the responses for each of the food insecurity indicators aggregated at the sub-national level. These maps (Figure 4, Figure 5, Figure 6, Figure 7, Figure 8, Figure 9, Figure 10 and Figure 11) show a wide heterogeneity both within and across countries. It is evident that food insecurity within these countries is spatially heterogeneous, suggesting that these challenges will require policies tailored to the specific situations in different parts of the each country. Increases in the prices of major food items and the different levels of increases within countries are reported to be the main reasons behind the heterogeneous sub-national level food insecurity. Supply chain disruptions leading to reduced access to staple food also resulted in differences in sub-national food insecurity. Import-dependent countries such as Djibouti also suffered from international supply chain disruptions, which led to knock-on effects in the domestic markets [38,39].

### 3.3. Socioeconomic Determinants of Food Insecurity

**Figure 12:**
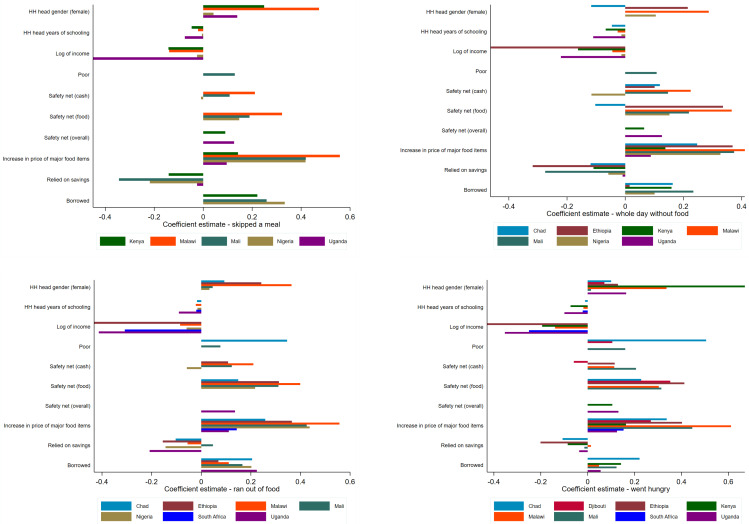
Regression coefficients—showing only statistically significant estimates.

Our analysis provides a first assessment on the social drivers of food insecurity during the pandemic and, as such, should be interpreted with caution. The results of our regressions are presented in Figure 12 (only statistically significant coefficients are shown) below (Detailed regression results are presented in the Appendix A; Table A1, Table A2, Table A3 and Table A4). Across all the specifications, female-headed households have a higher probability of suffering from food insecurity compared with male-led households, and this effect is particularly high in Malawi. While there are a few exceptions, the gender disparity is consistent across the four indicators of food insecurity that we study in this paper. Perhaps not surprisingly, we find that the chance of food insecurity is lower among households with relatively higher educated heads. However, this is still a critical finding because it suggests that education continues to play an important role even during a pandemic. What is particularly interesting, however, is that the strength of this difference varies considerably across countries, with the impact of education being most pronounced in Uganda.

Our results also suggest that higher-income households have a lower probability of suffering from food insecurity during the pandemic. Again, though this might be expected, the impact varies across countries, with the differential impact being strongest in Ethiopia and Uganda with respect to reducing food insecurity. For Djibouti and Mali, which provide data on poverty status of households instead, the regression estimates show that poorer households have a higher probability of suffering from food insecurity, with the probability of food insecurity due to poverty status of a household being higher in Chad.

Given the magnitude of the pandemic shock and the slow initial roll-out of safety nets from governments, it is perhaps not surprising that neither of the safety net policies (cash and food) appear to be consistently effective in reducing the probability of food insecurity across the four dimensions. However, the data do suggest that, at least in Nigeria and Djibouti, safety nets in the form of cash transfers have helped to reduce the probability of households skipping a meal and going hungry in Djibouti, and going without food for a whole day and running out of food in Nigeria. By contrast, food safety nets do appear to have reduced the probability of going without food for a whole day in just one country, Chad. Interestingly, in these three countries, the roll-out of assistance appears to have been relatively rapid, in as much as, by the time of the surveys, at least 20% of households in each of these three countries had been the recipient of at least one government safety net.

With regards to coping strategies, our results suggest that households that relied on their own savings during the pandemic have a lower probability of suffering from food insecurity. These results are consistent across the four food insecurity indicators for the countries in our analysis. This particular finding also implies an inequality impact as more wealthy and higher income households are more likely to have savings that they can use to make up for a loss in income or to increase expenditure.

## 4. Discussion

Female-headed households, the poor, and the less-formally educated appear to be suffering more in terms of food insecurity during the global COVID-19 pandemic. This aligns with much of the literature that addresses food insecurity more broadly. For example, even without economic crises and government-imposed restrictions, studies across lower-income countries have found examples of female-headed households generally experiencing higher levels of food insecurity [40]. In Nigeria, this has been attributed to female-headed households having fewer resources [17]. In Brazil, the 2009 National Household Survey revealed that female-headed households were 32% more likely to experience moderate food insecurity and 16% more likely to experience severe food insecurity compared with male-headed households [18]. In South Africa, rural male-headed households have been found to experience less food insecurity, due to their having more off-farm labour participation opportunities [19]. Perhaps particularly relevant for our study, ref. [20] found that during the 2007–2008 food price crisis, female-headed households experienced greater increases in food insecurity than male-headed households.

It is not immediately clear why female-headed households appear to have been particularly negatively affected by the COVID-19 pandemic in Malawi, across all four dimensions of food insecurity. Ref. [41] also found female-headed households to be more vulnerable than male-headed ones in Malawi, and attributed this difference to women having poorer access to and control of resources. However, many of the studies that purport to compare female- and male-headed households are actually comparing female-headed single-parent households with a group most likely dominated by households comprising married couples with children [40]. Recognising this, ref. [42] compared three types of households in Malawi, those with both female and male adults, and those with either female or either male adults. They found that food insecurity is highest in both female-only and male-only households compared with what they term dual-headed households. Ref. [43] similarly found that, more broadly, married couples are less likely than single people to experience food insecurity. A number of papers have found no statistical differences between the food insecurity status of female- and male-headed households. For example, in Bangladesh, little difference was found for indigenous households, one rationale being that women from these groups have more freedom to participate in the labour force [44]. Similar findings have been reported in three sites in Kenya, Tanzania, and Uganda [45].

In a meta-analysis of food security in Southern Africa covering 49 distinct studies, Ref. [46] found poverty, environmental stressors, and conflict to be the most important drivers of food insecurity. That poverty has been found to be closely linked to food insecurity, both in our analysis and in earlier literature, is perhaps not surprising. However, what is particularly interesting in our results is that the link between income and food insecurity appears to differ considerably among countries, with the effects in Chad, Ethiopia, and Uganda being particularly large. We have econometric evidence that households that were able to rely on their savings during the pandemic had a lower probability of suffering from food insecurity. This is supported by the fact that poorer households have suffered an income loss during the pandemic, and those in the lower-income quartiles are also more likely to suffer from food insecurity.

In common with earlier literature, for example, Ref. [24] in Sri Lanka, we find a small but significant link between years of formal education and food insecurity. However, Ref. [20] found that self-reported food insecurity actually increased more among more educated respondents in Africa during the food price crisis of the 2000s, suggesting that the relationship between food insecurity and formal education may be complex.

Our multi-country analysis shows that neither cash nor food safety nets are particularly effective in reducing the probability of food insecurity during the early stages of the pandemic. These results are unsurprising, given the magnitude of the shock. However, our data do tentatively suggest that cash transfers have been more effective than food transfers, particularly in Djibouti and Nigeria. Clearly specific circumstances matter, and indeed, the literature that addresses how and why particular transfers might be most appropriate for different types of households and under different circumstances is well developed and takes into account, for example, intra-household gender dynamics and how well food markets are functioning [26,27]. There are suggestions in the literature that stronger safety nets might be required, for example, basic income assurances, for vulnerable households living through crises such as the COVID-19 pandemic [6,47,48]. However, especially for lower-income countries, such interventions may be unrealistic.

Finally, we do of course recognise that the time when the survey data were collected matters. Food insecurity in lower-income countries tends to be closely linked to agricultural seasons, particularly where smallholder family farms dominate, and this is well documented in Africa [49]. This caveat not withstanding, overall, our findings suggest that whilst broadly the poor, the less educated, and female-headed households are likely to be experiencing, on average, relatively higher levels of food insecurity, there is considerable variation within and across countries. This suggests that for governments to most effectively tackle food insecurity linked to the pandemic, they need to be able to target their efforts, using data and analysis such as those we present here.

## 5. Conclusions

In this paper, we explored in detail key socioeconomic dimensions of food insecurity across nine countries in sub-Saharan Africa in 2020, during the early months of the COVID-19 pandemic. In contrast to papers that track one country’s experiences over time, we provided a snapshot of the extent to which households across the continent are having to deal with moderate and extreme levels of food insecurity during the pandemic, which appear to have been exacerbated by government efforts to stem the spread of the virus, combined with insufficient safety nets in place to mitigate the effects of the restrictions. We focused particularly on how poverty, education, and household composition differentially affect households’ experiences. Our analysis can help inform governments as to where their efforts need to be focused to reduce food insecurity. Even more so, we provided a baseline analysis of the available data so that, as more data are collected and reported, it will be possible to track the extent to which food security in these countries improves over time as well as the role of government policies and safety nets.

## Figures and Tables

**Table 1:** Food insecurity across countries during the pandemic (percent of respondents).

Country	Skip a Meal	Without Eating for a Whole Day	Run out of Food	Hungry but Did Not Eat
Chad	56.3	24.6	46.7	44.5
Djibouti	-	-	-	25.2
Ethiopia	-	10.9	21.9	17.1
Kenya	26.8	12.3	-	26.8
Malawi	64.0	26.6	49.6	54.7
Mali	13.7	3.7	9.8	9.5
Nigeria	72.9	24.5	56.9	-
South Africa	-	-	51.3	26.2
Uganda	36.9	11.2	25.7	27.2

## Data Availability

The data used in this paper are publicly available at https://microdata.worldbank.org/index.php/catalog/hfps. Accessed on 15 March 2021.

## Appendix A

**Table A1:** Determinants of food insecurity—skipped a meal.

	(1)	(2)	(3)	(4)	(5)
	**Nigeria**	**Kenya**	**Uganda**	**Mali**	**Malawi**
HH head gender (female)	0.042 ***	0.249 *	0.139 *	−0.012	0.473 ***
	(0.008)	(0.094)	(0.051)	(0.525)	(0.000)
HH head age	−0.034 ***	0.054	−0.038 ***	0.002	−0.021 ***
	(0.000)	(0.164)	(0.001)	(0.130)	(0.000)
HH head age-squared	0.0003 ***	0.0006	0.0003 ***	0.00002	0.0002 **
	(0.000)	(0.127)	(0.009)	(0.463)	(0.000)
HH head years of schooling	−0.005 **	−0.047 **	−0.075 ***		−0.021 ***
	(0.001)	(0.034)	(0.000)		(0.000)
Log of income	−0.025 ***	−0.142 ***	−0.450 ***		−0.139 ***
	(0.000)	(0.001)	(0.000)		(0.000)
Poor				0.129 ***	
				(0.007)	
Safety net (cash)	−0.009 ***			0.108 ***	0.211 ***
	(0.000)			(0.009)	(0.002)
Safety net (food)	0.147 ***			0.189 ***	0.322 ***
	(0.001)			(0.004)	(0.008)
Safety net (overall)		0.090 ***	0.126 ***		
		(0.000)	(0.000)		
Increase in price of major food items	0.418 ***	0.142 ***	0.096 ***	0.419 ***	0.558 ***
	(0.006)	(0.000)	(0.002)	(0.010)	(0.000)
Relied on savings	−0.218 ***	−0.141 ***	−0.025 ***	−0.344 ***	
	(0.001)	(0.008)	(0.000)	(0.014)	
Borrowed	0.333 **	−0.221 ***	−0.005	−0.259 ***	
	(0.001)	(0.008)	(0.114)	(0.004)	
Observations	3401	1787	3666	1370	1370

*p*-values in parentheses; *** *p* < 0.01, ** *p* < 0.05, * *p* < 0.10

**Table A2:** Determinants of food insecurity—went without eating for a whole day.

	(1)	(2)	(3)	(4)	(5)	(6)	(7)
	**Nigeria**	**Kenya**	**Uganda**	**Mali**	**Chad**	**Ethiopia**	**Malawi**
HH head gender (female)	0.105 ***	0.018	0.110	0.004	−0.117 ***	0.215 ***	0.287 ***
	(0.000)	(0.933)	(0.281)	(0.899)	(0.003)	(0.000)	(0.000)
HH head age	−0.006 ***	0.030	−0.014	−0.006 ***	−0.004 ***	−0.061 ***	−0.003
	(0.006)	(0.691)	(0.477)	(0.005)	(0.004)	(0.000)	(0.945)
HH head age-squared	0.00006 **	0.0006	0.0002	−0.00009 ***	0.000002 ***	0.0006 ***	−0.000003
	(0.014)	(0.472)	(0.373)	(0.008)	(0.005)	(0.000)	(0.938)
HH head years of schooling	−0.013 **	−0.067 **	−0.110 ***		−0.046 ***		−0.026 ***
	(0.000)	(0.045)	(0.000)		(0.005)		(0.000)
Log of income	−0.013 ***	−0.162 ***	−0.221 **			−0.463 ***	−0.044 *
	(0.000)	(0.005)	(0.027)			(0.000)	(0.059)
Poor				0.108 ***	0.135 ***		
				(0.000)	(0.002)		
Safety net (cash)	−0.116 ***			0.147 ***		0.101 ***	0.225 ***
	(0.000)			(0.007)		(0.005)	(0.000)
Safety net (food)	0.152 ***			0.219	−0.103 ***	0.336 **	0.366 ***
	(0.000)			(0.036) **	(0.000)	(0.030)	(0.003)
Safety net (overall)		0.065 **	0.126 ***				
		(0.047)	(0.000)				
Increase in price of major food items	0.327 ***	0.138 ***	0.088 ***	0.374 ***	0.247 ***	0.369 ***	0.411 ***
	(0.008)	(0.000)	(0.001)	(0.000)	(0.000)	(0.007)	(0.000)
Relied on savings	−0.058 ***	−0.109 ***	−0.009 ***	−0.275 ***	−0.119 ***	−0.318 **	
	(0.007)	(0.000)	(0.000)	(0.002)	(0.004)	(0.011)	
Borrowed	0.101 ***	0.159 ***	0.522	0.234 ***	0.163 ***	0.015 **	
	(0.004)	(0.001)	(0.147)	(0.003)	(0.000)	(0.042)	
Observations	3401	1787	2755	1372	12,227	14,409	1370

*p*-values in parentheses; *** *p* < 0.01, ** *p* < 0.05, * *p* < 0.10

**Table A3:** Determinants of food insecurity—ran out of food.

	(1)	(2)	(3)	(4)	(5)	(6)	(7)
	**Nigeria**	**South Africa**	**Uganda**	**Mali**	**Chad**	**Ethiopia**	**Malawi**
HH head gender (female)	0.033 ***	0.075	0.018	0.046 **	0.093 **	0.242 ***	0.364 ***
	(0.034)	(0.355)	(0.806)	(0.026)	(0.011)	(0.000)	(0.000)
HH head age	−0.032 ***	−0.076 ***	−0.042 ***	−0.003 *	0.009 ***	−0.043 ***	−0.023
	(0.000)	(0.000)	(0.001)	(0.094)	(0.006)	(0.000)	(0.000)
HH head age-squared	0.0004 ***	0.0009 ***	−0.0003 ***	−0.00002	−0.00008	0.0004 ***	0.0002
	(0.000)	(0.000)	(0.007)	(0.446)	(0.144)	(0.000)	(0.000)
HH head years of schooling	−0.015 **	−0.020 *	−0.090 ***		−0.017 ***		−0.022 ***
	(0.000)	(0.069)	(0.000)		(0.010)		(0.000)
Log of income	−0.059 ***	−0.308 ***	−0.413 ***			−0.432 ***	−0.085 ***
	(0.000)	(0.000)	(0.000)			(0.000)	(0.000)
Poor				0.077 ***	0.346 ***		
				(0.001)	(0.000)		
Safety net (cash)	−0.058 **			0.123 ***		0.108 ***	0.210 ***
	(0.034)			(0.006)		(0.001)	(0.000)
Safety net (food)	0.217 ***			0.311 ***	0.149 ***	0.313 ***	0.399 ***
	(0.000)			(0.000)	(0.000)	(0.007)	(0.002)
Safety net (overall)			0.136 ***				
			(0.009)				
Increase in price of major food items	0.437 ***	0.143 ***	0.111 ***	0.425 ***	0.258 ***	0.365 ***	0.557 ***
	(0.000)	(0.009)	(0.003)	(0.000)	(0.004)	(0.002)	(0.008)
Relied on savings	−0.144 ***		−0.208 **	−0.047 ***	−0.103 ***	−0.155 ***	
	(0.001)		(0.000)	(0.014)	(0.008)	(0.000)	
Borrowed	0.202 ***		0.224 **	0.166 ***	0.205 ***	0.069 ***	
	(0.008)		(0.032)	(0.000)	(0.000)	(0.001)	
Observations	3432	2526	3454	1372	12,213	14,409	1370

*p*-values in parentheses; *** *p* < 0.01, ** *p* < 0.05, * *p* < 0.10

**Table A4:** Determinants of food insecurity—went hungry.

	(1)	(3)	(4)	(5)	(6)	(7)	(8)	(9)
	**South Africa**	**Kenya**	**Uganda**	**Djibouti**	**Mali**	**Chad**	**Ethiopia**	**Malawi**
HH head gender (female)	−0.026	0.670 ***	0.164 **	0.071 ***	0.014	0.100 ***	0.129 ***	0.336 ***
	(0.771)	(0.003)	(0.026)	(0.000)	(0.497)	(0.007)	(0.007)	(0.000)
HH head age	−0.051 ***	0.053 *	0.017	0.007 ***	0.003 *	0.005 ***	−0.043 ***	−0.018
	(0.008)	(0.063)	(0.145)	(0.000)	(0.097)	(0.000)	(0.000)	(0.000)
HH head age-squared	0.0006 **	0.0007 **	−0.0001	−0.0001 ***	−0.00001	−0.000001 **	0.0004 ***	0.0002
	(0.018)	(0.039)	(0.306)	(0.000)	(0.597)	(0.047)	(0.000)	(0.000)
HH head years of schooling	−0.021 *	−0.073 *	−0.099 ***			−0.011 *		−0.018 ***
	(0.066)	(0.089)	(0.000)			(0.094)		(0.000)
Log of income	−0.250 ***	−0.194 ***	−0.353 ***				−0.429 ***	−0.139 ***
	(0.000)	(0.001)	(0.000)				(0.000)	(0.000)
Poor				0.105 ***	0.160 ***	0.505 ***		
				(0.000)	(0.000)	(0.000)		
Safety net (cash)				−0.059 ***	0.206 ***		0.115 **	0.113 ***
				(0.000)	(0.006)		(0.045)	(0.001)
Safety net (food)				0.352 ***	0.314 ***	0.228 ***	0.412 ***	0.303 *
				(0.000)	(0.000)	(0.004)	(0.000)	(0.090)
Safety net (overall)		0.105 ***	0.131 **					
		(0.000)	(0.040)					
Increase in price of major food items	0.153 ***	0.163 ***	0.125 ***	0.269 ***	0.446 ***	0.337 ***	0.402 ***	0.611 ***
	(0.003)	(0.005)	(0.009)	(0.000)	(0.001)	(0.000)	(0.004)	(0.004)
Relied on savings		−0.085 ***	−0.036 **		−0.014 ***	−0.107 ***	−0.201 ***	
		(0.001)	(0.000)		(0.008)	(0.000)	(0.000)	
Borrowed		0.142 ***	0.055 ***		0.123 ***	0.221 ***	0.118	
		(0.004)	(0.032)		(0.005)	(0.008)	(0.232)	
Observations	2526	1787	3409	1372	1372	12,040	14,409	1370

*p*-values in parentheses; *** *p* < 0.01, ** *p* < 0.05, * *p* < 0.10

## Appendix B. Immediate Economic and Health Impacts of COVID-19

Chad reported 1688 cases and 101 deaths related to COVID-19 up to the end of November 2020. Djibouti reported 5689 COVID-19 cases and 61 deaths at the end of November. Ethiopia reported more than 110,000 cases and more than 1700 deaths due to coronavirus by the end of November. Kenya reported 84,000 COVID-19 cases and nearly 1500 deaths until the end of November. Nairobi thus far reported 45% of all the cases in the country. Nigeria recorded the second highest number of infections in Sub-Saharan Africa, with 67,800 cases and 1200 deaths until the end of November. Malawi reported 6000 cases and 185 deaths related to COVID-19 at the end of November. In Mali there were 4762 COVID-19 cases reported and 160 deaths at the end of November. In Mali there was a 12.7% loss in jobs before the coronavirus onset while 4.9% of the family businesses reported a loss in income compared with the previous month. South Africa reported more than 792,000 cases of COVID-19 and nearly 22,000 deaths as of November 2020. Uganda reported 21,409 confirmed COVID-19 cases and around 200 deaths by the end of November 2020.
